# Guardians of the ocular surface: lessons learned from a challenging case of Langerhans cell histiocytosis with eyelid involvement

**DOI:** 10.5935/0004-2749.2024-0007

**Published:** 2024-09-16

**Authors:** Laura Goldfarb Cyrino, Andrea Santucci Cesar, Lia Zumblick, Juliana Kato, Patricia Picciarelli, Ruth Miyuki Santo

**Affiliations:** 1 Ophthalmology Department, Universidade de São Paulo, São Paulo, SP, Brazil; 2 Cornea and External Eye Diseases Sector, Ophthalmology Department, Universidade de São Paulo, São Paulo, SP, Brazil

**Keywords:** Eyelid diseases/diagnosis, Histiocytosis, Langerhans cell/diagnosis, Orbital diseases, Ectropion, Dry eye syndromes, Erdheim-Chester disease/drug therapy, Human, Female, Case reports

## Abstract

Langerhans cell histiocytosis comprises a heterogeneous range of clinical manifestations secondary to clonal proliferation of histiocytes, characterized by the accumulation of these cells in various organs and tissues. The ophthalmological component commonly involved is the orbit. Herein, we report a rare case of Langerhans cell histiocytosis with eyelid involvement, which resulted in severe ocular surface complications, which subsequently significantly impacted the patient’s quality of life. This case report highlights the fact that despite being rare, Langerhans cell histiocytosis should be included in the differential diagnosis of eyelid lesions. Furthermore, a multidisciplinary approach with a systemic overview is crucial for managing the ocular complications.

## INTRODUCTION

Langerhans cell histiocytosis (LCH) is the clonal proliferation of histiocytes. The systemic manifestations of LCH depend on the affected organ^(^1^)^. In LCH, ocular and orbital involvement is uncommon and exclusive eyelid involvement is rare^(^2^,^3^)^. Thus, herein, we aimed to present a rare presentation of LCH with eyelid involvement, which resulted in severe ocular surface complications.

## CASE REPORT

**Figure 1:**
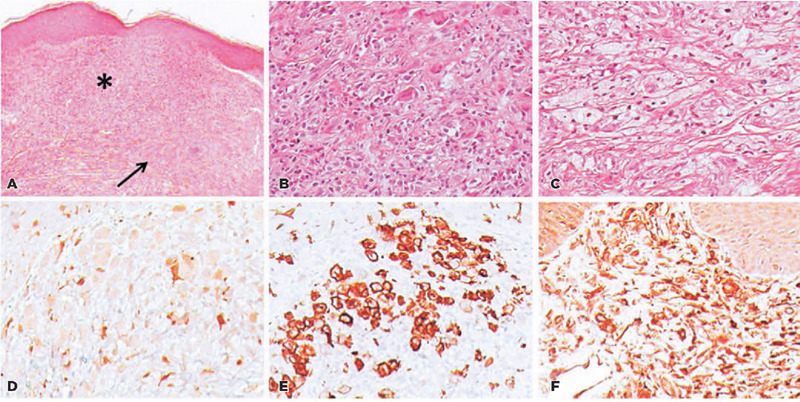
Histopathological analysis of the specimen. Hematoxylin and eosin staining revealed the following: A) diffuse infiltration of the dermis by histiocytes (*) and areas of fibrosis (arrow); B) typical Langerhan’s histiocytes with epithelioid cytoplasm; and C) histiocytes with large and xamtomized cytoplasm (component of Erdheim-Chester Disease) (20x magnification). Immunohistochemistry for D) S100, E) CD-la, and F) CD-68.

A 53-year-old female presented with a single, solid, gradually progressive nodule on the upper left eyelid margin in 2005. In 2007, the lesion was excised at our center’s Department of Dermatology. Histopathological analysis of the specimen revealed histiocytes with eosinophilic cytoplasm and immunohistochemical positivity for S-100 protein, CD-1a, CD-68, and CD-34, which was of LCH. Furthermore, histiocytes with large and xamtomized cytoplasm were present, which indicated a component of Erdheim-Chester Disease (ECD) (Figure 1). At the time, the patient denied having other similar lesions on her body.

**Figure 2:**
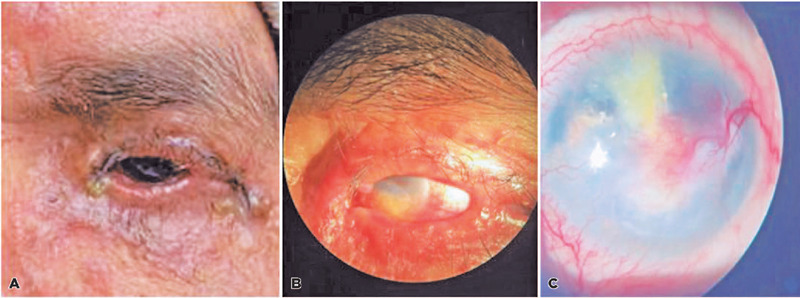
Slit lamp examination of the OS.

In 2021, the patient was referred to the Department of Ophthalmology for the first time. The patient presented with incomplete eyelid closure of the left eye (OS), which lead to exposure of the ocular surface. The patient reported that the symptoms started after the diagnostic excision of an eyelid nodule. The patient was diagnosed with exposure keratitis due to incomplete intraocular closure, and a definitive tarsorrhaphy was performed (Figure 2).

In July 2022, the patient developed a corneal ulcer in the OS, which caused corneal leukoma, visual impairment, and severe pain. Slit lamp examination of the OS revealed a temporal tarsorrhaphy, incomplete ocular closure, paracentral leukoma with inferior neo-vascularization, and diffuse punctate keratitis (Figure 2). The visual acuity was 0.90 in the right eye (OD) and light perception in the OS. The patient was diagnosed with exposure keratitis in the OS with severe ocular sequelae due to the incomplete eye closure caused by the diagnostic eyelid surgery. Treatment was initiated in the OS with 0.15% sodium hyaluronate lubricant and an ophthalmic ointment which contained 10,000 IU/g of retinol acetate, 25mg/g of amino acids, 5mg/g of methionine, and 5mg/g of chloramphenicol.

**Figure 3:**
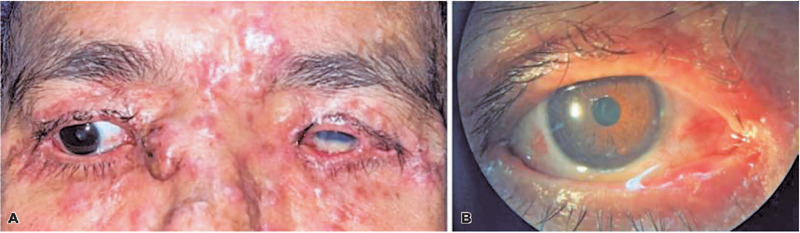
Slit lamp examination of the OD.

After eight months, the patient experienced an improvement in visual acuity and decrease in ocular pain. However, the patient developed excessive tearing in the OD and a new progressively growing nodule in the right epicanthus, which was associated with multiple nodules throughout the body. This indicated disease progression. Slit lamp examination of the OD revealed a nodule in the epicanthal region, multiple periocular nodules, dry periocular skin, a significant ectropion, calm conjunctiva, and a normal and transparent cornea (Figure 3).

Treatment was prescribed to improve the protection of the OD. The patient is currently being followed up with the hematology and dermatology departments.

## DISCUSSION

Histiocytosis is a group of disorders characterized by the abnormal accumulation of cells that are believed to be derived from dendritic cells or macrophages^(^4^)^. Histiocytosis can be classified into in the following three categories: Langerhans cell, non-Langerhans cell-related (including ECD), and malignant^(^5^)^. However, recent studies have demonstrated an association between LCH and ECD. Approximately 20% of the patients with LCH have an ECD component^(^4^,^6^)^. The revision of the histiocytosis classification^(^4^)^ revealed similarities between LCH and ECD in terms of histology, molecular alterations, and clinical presentation. Thus, a mixed LCH and ECD variant was proposed. This explains the overlap of features founded in our histopathological analysis.

LCH can be classified into the following two spectra on the basis of the involvement of organs and systems: single and multisystem. In 60% of the cases, a single system is affected, including bones and skin. The diagnosis is typically made in childhood^(^1^)^. Histiocytosis with palpebral involvement is extremely rare, with only 16 reported cases^(^2^,^3^)^. Herein, we described an atypical case of LCH with a component of ECD, in which the first clinical sign was palpebral involvement. Eyelid tumors are among the most common diseases occurring in the periocular area. However, LCH is rarely occurs here. Thus, the diagnosis may be delayed if a biopsy is not performed^(^7^)^. In addition to making the diagnosis, the management of the ocular surface complication due to both the diagnostic procedure and disease progression on the eyelid was challenging. The biopsy of the OS palpebral lesion was absolutely necessary to determine the etiology. However, it resulted in incomplete eyelid closure and subsequent exposure of the ocular surface. Furthermore, the disease progression, from a single nodule to multiple periocular nodules contributed to increased exposure of the ocular surface. This in turn caused eyelid retraction, lagophthalmos, and an ectropion.

Ectropion of the lower eyelid typically arises due to poor eyelid closure^(^8^)^. When the cornea is not adequately protected by the eyelid due to a loss of contact, the secretion produced by the Meibomian glands ceases, which leads to stasis and meibomitis. This cascade of events, results in dry eye syndrome, which is characterized by redness, excessive tearing, and a sensation of a foreign body in the eye^(^9^)^. In extreme cases of dry eye, exposure keratitis can progress to ulceration and visual loss^(^8^)^, which was seen in our patient. Furthermore, our patient developed a cicatricial ectropion due to the biopsy in the OS and anterior lamella shortening, which was related to dermatological lesions.

The treatment of dry eye in ophthalmic practice is extremely challenging. According to the Dry Eye Workshop 11 classification, our patient’s dry eye would be categorized as evaporative dry eye due to the eyelid closure disorder. The proposed treatment of dry eye depends on the etiology and severity of the disease^(^9^)^. In our patient, the progression to an incomplete eyelid closure in the OS justified the performance of tarsorrhaphy to reduce corneal exposure. Furthermore, the combination of lubricant eyedrops and vitamin ointment was advised to improve the lubrication and protection of the ocular surface.

The disease progression and surgical procedure led to ocular surface complications which were challenging to manage. Although the biopsy of an eyelid lesion is crucial for determining the etiology, the ocular surface should be protected. Furthermore, a multidisciplinary approach is required for managing heterogeneous cases^(^10^)^.

In conclusion, we have described a revised molecular pathology and the clinical features of LCH, especially ocular involvement, in this report with the aim to make ophthalmologists aware of atypical LHC presentations. Our experience with this case highlights the eyelid’s key role in protecting the ocular surface.
